# Cervical Fracture/Subluxation in a Patient with a Prior C2-Sacrum Fusion: Case Report and Review of Literature

**DOI:** 10.7759/cureus.888

**Published:** 2016-11-22

**Authors:** Yi-Ren Chen, Alvin Y Chan, Kevin K Kumar, Anand Veeravagu

**Affiliations:** 1 Department of Neurosurgery, Stanford University Medical Center; 2 Medical College of Wisconsin

**Keywords:** adjacent level disease, scoliosis

## Abstract

Traumatic injury to an adjacent segment of a previously fused spine is a rare complication of scoliosis surgery. The adjacent spinal segments may be more vulnerable to traumatic fracture or dislocation due to increased strain. We present a patient with prior C2 to sacrum fusion who suffered a C2 fracture/dislocation after falling. A 52-year-old female with a previous C2 to the sacrum fusion for idiopathic scoliosis presented with severe and progressively worsening neck pain after multiple falls. Imaging showed anterior displacement of the C2 vertebral body, fracture of C2, and anterior subluxation of the C1-2 complex on C3. The patient underwent posterior occiput to cervical fusion and reduction of the C1-C2 complex. Our case describes a potential complication of long-segment fusion. Adjacent segments may be more prone to fracture-dislocation because of increased intradiscal pressure and strain. Clinicians should have a high suspicion of fractures in patients with prior spinal fusions in the setting of trauma.

## Introduction

Traumatic fracture or dislocation of adjacent spinal segments is a rare but severe complication of a previously fused spine for idiopathic scoliosis. Fusion of a spinal segment increases strain on adjacent segments. These changes can be characterized by two major criteria: radiographically-evident adjacent segment degeneration and the new onset of clinical symptoms defined as adjacent segment disease. Furthermore, idiopathic scoliosis patients with thoracic to sacrum fusions have been shown to suffer higher incidence and severity of degeneration in the cervical spine compared to those without fusion [1]. Despite this risk of degenerative adjacent level disease, acute traumatic injury to adjacent vertebral segments in scoliosis patients is a relatively rare occurrence.

We present the case of an idiopathic scoliosis patient with a prior C2 to sacrum fusion who suffered a C2 fracture/ subluxation after falling and a review of the literature of traumatic fracture or dislocation adjacent to prior cervical fusions.

## Case presentation

This is a case of a 62-year-old female who underwent a C2 to the sacrum fusion previously for progressive and worsening idiopathic scoliosis. Her prior surgeries were performed in multiple stages due to proximal junctional kyphosis at various levels. She suffered multiple falls down stairs and presented to the emergency department with severe and worsening neck pain. She also reported bilateral upper extremity numbness and tingling accompanied by balance difficulties while ambulating. Prior to this series of falls, she was living and functioning independently at home.

**Figure 1:**
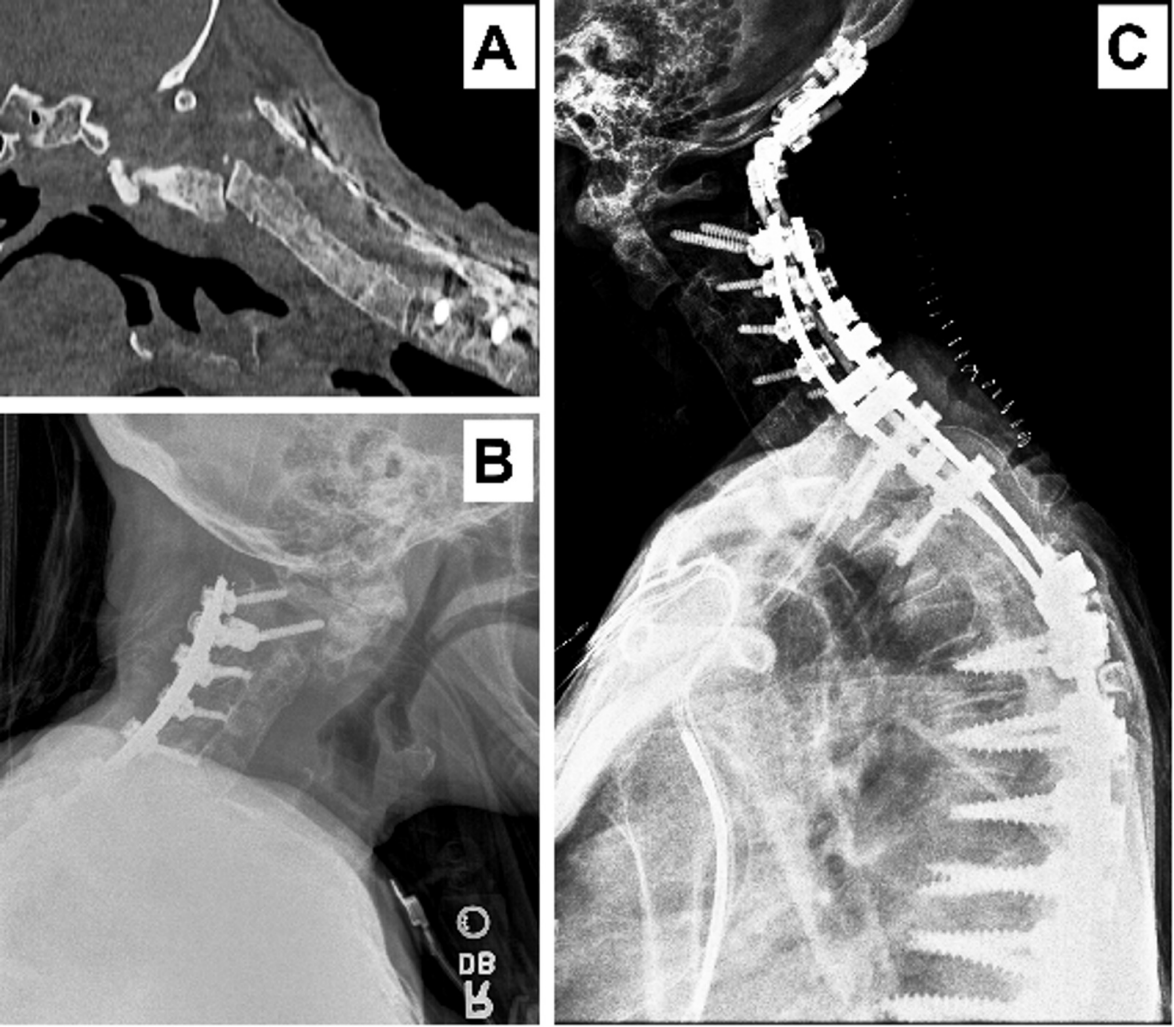
Imaging Panel A and B—Imaging showing the fracture and subluxation; Panel C—Imaging showing occiput to C3 extension of fusion.

On examination, our patient had full strength in all muscle groups with a positive right Hoffman’s reflex and 2+ reflexes otherwise. Computed tomography (CT) scan of the neck demonstrated the following: (1) a 7 mm anterior displacement of the C2 vertebral body, (2) multi-comminuted and angulated fracture of C2, (3) anterior subluxation of the C1-2 complex on C3, and (4) subluxation of the foramen magnum (Figure 1AB). She was unable to undergo magnetic resonance imaging (MRI) due to an implanted defibrillator device.

Given the patient’s significant pain and deformity of the C1-2 complex, a posterior occiput to cervical fusion and reduction of the subluxed C1-C2 complex was performed (Figure 1C). There were no complications with the procedure, and the patient retained full strength with improvement alignment on postoperative x-ray imaging. 

## Discussion

We present a scoliosis patient with a prior C2-sacrum fusion who suffered a C2 fracture/subluxation after a fall. There are few additional reports in the published literature of traumatic fractures adjacent to a fused cervical spine. Mac Millan and Stauffer outlined four such cases in patients with prior upper-level fusions (C2-C3 or C3-C4) and subsequent type 3 odontoid fractures [2]. In addition, two of the patients had posterior C1 arch fractures. In the presented case, the traumatic force experienced by the patient likely affected adjacent levels more since fused segments cannot flex or extend, thus transferring all the force to the adjacent level.

A review of the literature identified four additional cases of fractures adjacent to prior cervical fusions (Table 1). Mechanisms included motor vehicle accidents and falls, resulting in two injuries above the fused segments and two below [3-6]. However, the presented case has some notable differences from these previous reports. First, none of them described patients with long fusions to treat scoliosis. Yoshihara, et al. described the longest fusion, extending from C3 to C7 [5]. We suspect that the length of our patient’s fusion could have contributed to her traumatic injury, as scoliotic patients with long fusions to the sacrum generally have high complication rates [7]. Secondly, previous studies have not described a fracture or dislocation as high as C2. The highest fractures or dislocations described were C4-C5 facet dislocations [3-4]. Mac Millan and Stauffer reported four patients with C2 fractures, but those were adjacent to single-level fusions [2].

**Table 1:** Summary Summary of cases describing acute traumatic injury adjacent to cervical spinal fusions.

Author	Year	Patient	First Pathology	Fusion	Trauma	Second Pathology	Treatment
Whitehill, et al. [3]	1987	21 y.o. male	C5-C6 posterior spinous process space dislocation	Posterior interspinous arthrodesis (C5-C6)	Motor vehicle crash	C4-C5 bilateral facet dislocation	Open reduction and fusion at C4 to C5
Mac Millan and Stauffer [2]	1991	4 patients with fusions C3-C4 or above; 8 patients with fusions C4-5 or below	N/A	Surgical (anterior or posterior fusion); congenital (Klippel-Feil type 2, type 3); degenerative ankylosis	N/A	Motor vehicle accident; bicycle accident; unclear	Fusion; conservative treatment (external immobilization)
Yoshihara, et al. [5]	2011	61 y.o. female	Progressive kyphosis with collapse of the C5 vertebral body	Anterior cervical discectomy and fusion (C3 to C7)	Fall	C7 and T1 vertebral body fracture, subluxation of C7-T1 facets, C7 spinous process fracture	Posterior fusion from C7 to T2; T1 vertebral body removal replaced with cage/bone graft
Raizman, et al. [4]	2012	55 y.o. female	C5-C6 disc herniation	Anterior cervical discectomy and fusion (C5-C6)	Motor vehicle crash	C4-C5 unilateral facet dislocation with posterior disc herniation	Decompression and reduction of herniation with anterior plate at C4-C5
Yokoyama, et al. [6]	2016	79 y.o. male	C5-C6 degenerative cervical spine disease	Anterior cervical discectomy and fusion (C5-C6)	Fall	Severe anterior dislocation of C6-C7; spinal cord edema	Laminectomy of C5-T1 and posterior fusion of C6-C7

While there is no standard protocol for management of adjacent level disease in cervical fusions, surgery is typically indicated after trauma. Four cases, including ours, were treated with various types of fusions [3, 5-6], one of which included an anterior plate fixation [4]. Notably, patients with adjacent C2 fractures were treated with external immobilization rather than surgery to protect cervical motion [2]. However, the long-term outcomes for both surgical or conservative treatments are unknown.

We hypothesize our patient had asymptomatic adjacent level pathology at C2 that rendered the region hypersusceptible to injury. Studies in cadaveric models have demonstrated that immobilization of C5-C6 increased motion in adjacent unfixed regions [8]. Such increased motion could result in higher intradiscal pressure. Cervical fusion may also increase shear and longitudinal strain on adjacent intervertebral discs. Preserving motion via discectomy instead of fusion has been shown to slow progression of symptomatic and radiologic adjacent disc disease [9]. Conversely, patients with cervical fusions likely develop adjacent disc pathology faster than non-fusion patients, although they may remain clinically asymptomatic. Thus, further investigation of the impact of segment fixation on the biomechanics of traumatic injuries in adjacent levels is of considerable value to this patient population.

## Conclusions

We describe a C2 fracture/dislocation as a potential complication of long fusion to the sacrum, potentially caused by increased vulnerability due to asymptomatic adjacent disc pathology. Clinicians should be vigilant of adjacent segment fractures or dislocations when patients with fusions experience acute trauma.
